# X-ray beam diagnostics at the MID instrument of the European X-ray Free-Electron Laser Facility

**DOI:** 10.1107/S1600577524001279

**Published:** 2024-04-08

**Authors:** Ulrike Boesenberg, Gabriele Ansaldi, Alexander Bartmann, Lewis Batchelor, Felix Brausse, Jörg Hallmann, Wonhyuk Jo, Chan Kim, Birthe Klein, Iker Lobato, Wei Lu, Johannes Möller, Ilia Petrov, Angel Rodriguez-Fernandez, Andreas Schmidt, Markus Scholz, Roman Shayduk, Konstantin Sukharnikov, Alexey Zozulya, Anders Madsen

**Affiliations:** a European X-ray Free-Electron Laser Facility, Holzkoppel 4, 22869 Schenefeld, Germany; RIKEN SPring-8 Center, Japan

**Keywords:** X-ray free-electron lasers, beam diagnostics, bent-diamond-crystal spectrometer, Materials Imaging and Dynamics instrument, multipurpose diagnostic end-station

## Abstract

The Materials Imaging and Dynamics (MID) instrument at the European X-ray Free-Electron Laser Facility (EuXFEL) is equipped with a multipurpose diagnostic end-station (DES) at the end of the instrument. The imager unit in DES is a key tool for aligning the beam to a standard trajectory and for adjusting optical elements such as focusing lenses or the split-and-delay line. Furthermore, the DES features a bent-diamond-crystal spectrometer to disperse the spectrum of the direct beam to a line detector. This enables pulse-resolved characterization of the EuXFEL spectrum to provide X-ray energy calibration, and the spectrometer is particularly useful in commissioning special modes of the accelerator. Together with diamond-based intensity monitors, the imager and spectrometer form the DES unit which also contains a heavy-duty beamstop at the end of the MID instrument. Here, we describe the setup in detail and provide exemplary beam diagnostic results.

## Abstract 2

The multipurpose diagnostic end-station (DES) at the Materials Imaging and Dynamics (MID) instrument at the European X-ray Free-Electron Laser Facility (EuXFEL) is described in detail and some exemplary beam diagnostic results are provided. Among other features, the DES is a key tool for alignment and the integrated bent-diamond crystal spectrometer enables pulse-resolved spectral characterization of the EuXFEL X-ray beam.

## 1. Introduction

The European X-ray Free-Electron Laser facility (EuXFEL) offers unique X-ray beam parameters with photon energies currently up to 25 keV, a maximum repetition rate of 4.5 MHz and up to 2700 pulses provided in pulse train bursts of 10 Hz repetition rate (Tschentscher *et al.*, 2017 ▸; Decking *et al.*, 2020 ▸). In addition to the delivery of self-amplified spontaneous emission (SASE) X-rays, a number of special operation modes such as hard X-ray self-seeding (HXRSS) (Liu *et al.*, 2023 ▸), two-color mode, ultrashort pulses and higher photon energies are under development. Although most of the photon-diagnostic devices for setup and tuning are located in the photon tunnels (Grünert *et al.*, 2019 ▸), careful characterization of the beam properties close to the sample interaction point remains important for the experiments. Despite the very small natural divergence of (SASE) radiation of a few microradians (Sinn *et al.*, 2011 ▸), the beam properties at the sample position may vary slightly from those measured in the tunnel due to the long beam transport path of about 1000 m between the last undulator and the experimental station. While single pulse full-wavefield characterization is extremely challenging at megahertz repetition rates, important information about the spectral distribution and intensity can be extracted more easily using a bent-crystal spectrometer and diamond-based solid-state ion chambers, respectively. The operation of such devices for hard X-ray free-electron laser (XFEL) beam characterization has been demonstrated previously (Zhu *et al.*, 2012 ▸; Inubushi *et al.*, 2012 ▸; Boesenberg *et al.*, 2017 ▸; Roth *et al.*, 2018 ▸). Due to the stochastic process of SASE, the intensity as well as the photon energy distribution varies from pulse to pulse. A relatively homogeneous intensity distribution and small beam pointing jitter over the pulse train can be achieved by optimized accelerator settings. Similarly, a variation or chirp in the spectral energy distribution over the train can be corrected, given that suitable pulse-resolved diagnostics are available. Because of the heat-load arising from the unprecedented high repetition rate and intensity at EuXFEL, diamond is the material of choice for devices inserted in the direct beam (Uhlén *et al.*, 2013 ▸).

**Figure 1:**
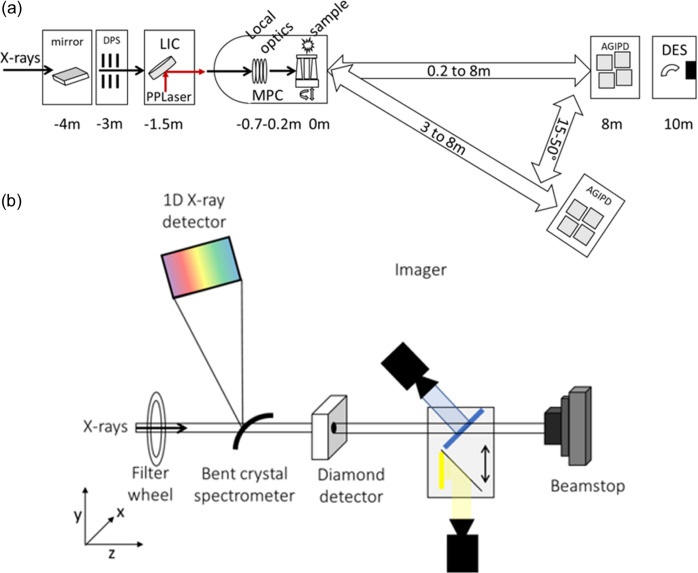
Schematic setup of the key devices constituting (*a*) the MID instrument and (*b*) the DES in particular. X-rays travel along the *z* direction. All components in the DES are installed in two connected vacuum chambers at the end of the MID beamline.

Here, we describe the available diagnostic tools with exemplary results for characterizing the X-ray beam on the inter- and intra-train level at the Materials Imaging and Dynamics (MID) instrument of EuXFEL (Madsen *et al.*, 2021 ▸). A diagnostics end-station (DES) with the desired functionality has been built and is mainly designed for use during setup, alignment and tuning, but can also be employed parasitically in combination with (semi-)transparent samples and setups. The limited space available towards the end of the experimental hutch of MID favors a compact design of DES with a footprint of the full device of approximately 0.8 m × 0.9 m × 2.5 m (width × length × height). It consists of four key devices: (1) a spectrometer to be inserted in the direct beam, (2) diamond-based intensity monitors, (3) an imager unit and (4) a beam stop. In addition, a filter wheel is included to provide beam attenuation or thin metal foils for photon energy calibration. Fig. 1 ▸(*a*) sketches the overall layout of the MID experimental hutch whereas Fig 1 ▸(*b*) illustrates the basic DES setup, located about 10 m downstream of the sample interaction point. Examples for the different applications and use cases are given, such as variation of the number of pulses per train, new machine settings under development such as HXRSS and ultra-short pulses, or alignment of special devices at the MID instrument such as the X-ray split-and-delay line (SDL).

## 2. Experimental setup and measurements

### 2.1. Overall setup

**Figure 2:**
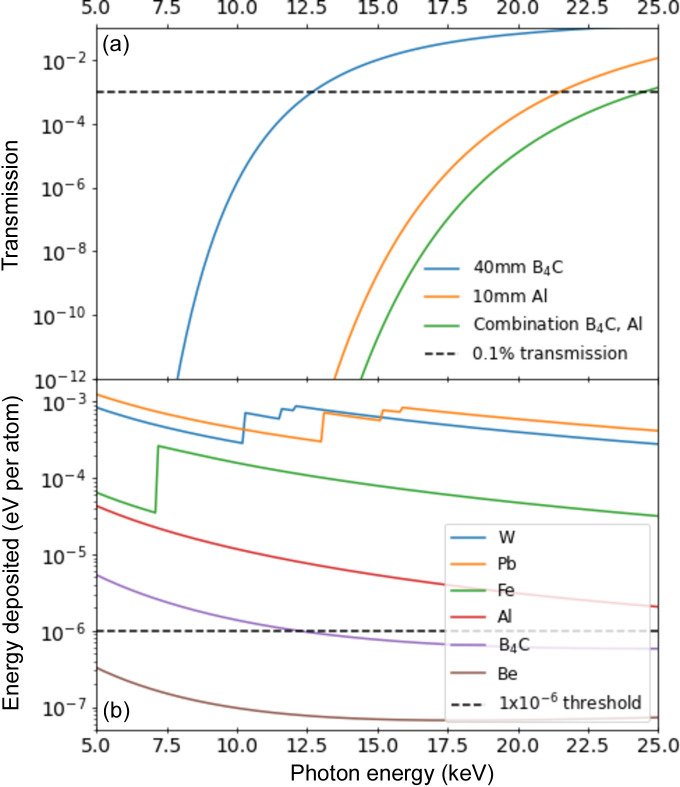
(*a*) Calculated transmission of the beamstop materials in a range from 5 keV to 25 keV (Henke *et al.*, 1993 ▸). (*b*) Energy deposited per atom in a single pulse. For simplicity our calculation used 10^10^ photons spread over a beam of 1 mm × 1 mm. In (*b*) we included the approximate ablation threshold for a focused beam of 10 µm × 10 µm and 10^12^ photons per pulse at 1 × 10^−6^.

The design of the MID optics implies a variable beam height over the floor in the experimental hutch, depending on the use of X-ray monochromators and other optical elements (Madsen *et al.*, 2021 ▸). Hence, to preserve the mutual alignment of the subdevices, including the arched rail system for the spectrometer detector, the DES unit is mounted on a vertically motorized table to account for the varying beam positions. Horizontal adjustments can be made by sliding the chambers on rails. The unit is separated into two vacuum chambers, where one houses the spectrometer as well as the intensity monitors and the filter wheel. The second chamber contains the imager unit and the layered beamstop with 40 mm of B_4_C followed by 10 mm of Al and 22 mm of steel (vacuum chamber flange). All subdevices are equipped with in-vacuum motors. Fig. 2 ▸(*a*) shows the calculated beamstop transmission of photons in the energy range from 5 keV to 25 keV, both for 40 mm B_4_C and for 10 mm Al as well as a combination of the two materials. Even at 25 keV, the combined transmission does not exceed 0.1%. The high peak intensity and repetition rate of the EuXFEL beam may lead to absorption of photons above the ablation threshold in high-*Z* materials typically used for beamstops. Fig. 2 ▸(*b*) shows the calculated energy deposited per atom and single pulse for an intensity of 10^10^ photons spread over a beam of 1 mm × 1 mm (Schoonjans *et al.*, 2011 ▸; Madsen *et al.*, 2013 ▸) for Pb, W, Fe, Al, Be and B_4_C. Typically, a value of approximately 1 eV per atom is to be considered the ablation threshold, depending on the repetition rate and photon beam energy. The curves clearly show the strong interaction of high-*Z* materials with (hard) X-rays and the potential risk with respect to their use as beamstop materials, especially for tightly focused and unattenuated beams. In the case of 10^12^ photons per pulse with 10 µm × 10 µm beam size, the *y* axis in Fig. 2 ▸(*b*) must be multiplied by 10^6^, which brings even B_4_C into the danger zone of ablation (∼1 eV), so precautions must be taken not to focus the beam on the beamstop. In more detail, this has been modeled and experimentally investigated by Yang *et al.* (2022 ▸), taking also the megahertz repetition rate into account. The beamstop is an integral part of the instrument safety, as significant ablation of material would affect the beamline vacuum system and hence operation. We have designed a layered beamstop consisting of 40 mm B_4_C and 10 mm Al which are sandwiched to a standard stainless steel vacuum flange (22 mm thickness) with graphite foils to improve the heat conductivity. The idea is to position the ablation hard and light materials first to take the top of the beam power, followed by more dense materials for absorption purposes. A future upgrade to harder X-rays (∼70 keV) at SASE-2 (Casalbuoni *et al.*, 2023 ▸) will require adding to the beamstop sandwich (*e.g.* by introducing graphite and lead layers).

### 2.2. Intensity monitor

**Figure 3:**
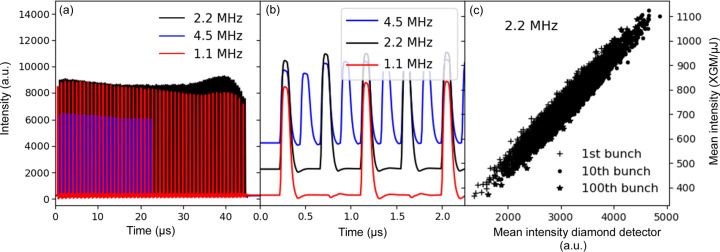
Pulsed-resolved intensity data at different repetition rates (4.5 MHz, 2.25 MHz and 1.1 MHz) taken with diamond-based intensity monitors at MID. (*b*) Zoomed-in image of (*a*) showing that the detector response is fast enough to accurately measure pulse intensities at a 4.5 MHz repetition rate. The duration of the pulse signal (approximately 150 ns FWHM) reflects the speed of the electronics and detector, not the X-ray pulse duration, which is less than 100 fs. (*c*) Correlation with the upstream gas monitor for the 1st, 10th and 100th pulse (2.2 MHz pulse repetition rate).

An X-ray gas monitor (XGM) (Maltezopoulos *et al.*, 2019 ▸) is installed for intensity characterization in the SASE-2 branch at approximately 210 m downstream of the undulator source point, but upstream of all optical devices such as mirrors, focusing optics and attenuators. The XGM output (beam energy per pulse) usually provides a good correlation with the intensity at the sample position, but insertion of additional optical devices such as a monochromators or X-ray lenses naturally disturb this correlation. Here, an intensity measurement closer to the sample is advantageous to normalize the scattered photons to the incoming intensity *I*_0_. The intensity monitors must be small enough to fit into the experimental setup, fast enough to cope with a repetition rate of 4.5 MHz and highly transmissive. Diamond-based solid-state detectors are well known from synchrotron radiation applications, where they are used as beam-position monitors (BPMs) (Shu *et al.*, 1998 ▸; Bergonzo *et al.*, 1999 ▸; Desjardins *et al.*, 2018 ▸). A diamond-based solid-state ionization chamber is located in the DES. The diamond plate has a thickness of 40 µm and is coated with 250 nm-thick layers of Be on each side that act as electrodes. A moderate bias voltage not exceeding 100 V is applied over the electrodes and the outgoing signal is read out by a fast analog to digital converter (StruckSIS8300). The detector is described in detail in an earlier publication by Roth *et al.* (2018 ▸). Fig. 3 ▸(*a*) shows the intensity distribution measured at different repetition rates (4.5 MHz, 2.2 MHz and 1.1 MHz) over the pulse train at a photon energy of 8.8 keV. In Fig. 3 ▸(*b*) the beginning of the train is enlarged [zoomed-in image of Fig. 3 ▸(*a*)]. The signal is clearly resolved for each individual X-ray pulse at all selected repetition rates.

In Fig. 3 ▸(*c*), correlation plots for the pulse intensity of the XGM versus the diamond detector is shown for the 1st, 10th and 100th pulse for the 2.2 MHz data, which show good correlation for these X-ray beam parameters. For long pulse trains, especially at higher intensities, accumulation of charge has been observed leading to a decrease in signal over the pulse train. A detailed characterization of the linear range for these detectors is still outstanding due to the large number of variables such as photon energy, intensity, bias voltage, electronic attenuation and beam size. A research and development project is ongoing to further improve and extend the capabilities of diamond detectors for megahertz XFEL applications (Çonka Yildiz *et al.*, 2023 ▸).

### 2.3. Imager

**Figure 4:**
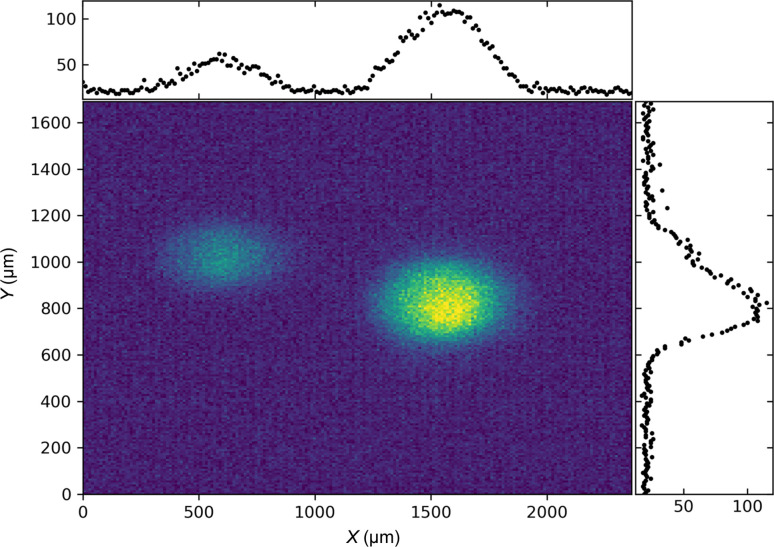
Two separated beams created by the SDL situated in the optics hutch about 20 m upstream of the DES.

The imager unit consists of two parts, providing high- and low-resolution images, respectively. The low-resolution option is equipped with a 20 µm B-doped diamond screen making an angle of 45° with respect to the direct beam. Hence it can remain in the beam also for a large number of pulses and high intensity without suffering from beam damage. An out of vacuum objective (KOWA LM50JC10M) in combination with a video camera (BASLER A2500-14gm) is used to achieve a field of view of approximately 15 mm × 15 mm and a pixel size of 12 µm × 8.5 µm, providing an image with 10 Hz repetition rate, hence averaging over one full pulse train. It allows a reproducible alignment of the beam as well as pointing corrections and is a valuable tool for the final alignment of the instrument components such as nanofocusing lenses, bent-diamond spectrometer, the SDL and for general sample alignment. As an example, Fig. 4 ▸ shows the two beam spots created by the SDL located approximately 20 m upstream of the imager (Lu *et al.*, 2016 ▸, 2018 ▸) during the alignment procedure; in this case the image is slightly out of focus. The two beams overlap at the sample position but the slight difference in pointing angles allows us to measure two separated beams in the DES and determine the angle; in the example in Fig. 4 ▸ it is 100 µrad. The variation in position and intensity permits monitoring of the long-term position stability of the SDL crystals and the angle of the impinging beam. Monitoring and correcting the angular mismatch is important, for example, in double-pulse X-ray speckle visibility spectroscopy experiments (Sun *et al.*, 2020 ▸).

Still under commissioning is a high-resolution beam imaging option based on a 25 µm-thick YAG:Ce screen in combination with a mirror, objective (currently KOWA LM50JC10M) and a camera (BASLER A2500-14gm). The target design is an approximate field of view of 2 mm × 2 mm and a pixel size of about 2 µm with a new objective and camera.

Both screens are mounted on a plate supported by three translation positioners (SmarAct) in the *x*, *y* and *z* directions for alignment and to switch between the two imaging options.

### 2.4. Spectrometer

Bent crystals have proven to be powerful tools in the characterization of SASE beams at hard XFEL sources allowing a very compact design (Zhu *et al.*, 2012 ▸; Boesenberg *et al.*, 2017 ▸; Kujala *et al.*, 2020 ▸; David *et al.*, 2021 ▸). At the DES, to cover the required photon energy range, a holder for a number of different bent crystals is available, comprising different materials and crystal orientations which can be mounted next to each other on the rotation axis of a high-precision piezo stage (NANOS Instruments GmbH). It has a measuring and positioning precision of 12000000 counts per rotation which corresponds to an accuracy of 0.5 µrad. Coarse alignment and positioning of crystals in the beam is achieved by a combination of translation motions perpendicular to the beam situated beneath the spectrometer holder. Rotation around the beam axis (roll) is provided by a cradle, allowing for adjustments of the diffracted signal to pass the Kapton window and enter the detector. Two detectors, a Gotthard-I X-ray linear (1D) detector with 1280 pixels of 50 µm pitch (1.2 mm wide) and a 2D imager with a 25 µm YAG screen, are mounted on a circular rail with a distance of about 1 m from the bent diamond crystal. With the current objective (KOWA LM50JC10M), a pixel size of approximately 6 µm × 6 µm is achieved with the 2D imager. The angular range of the Gotthard detector on the rails covers approximately 45° to 110°, allowing the collection of spectra from a bent diamond crystal in (110) orientation using either the C(220) or the C(440) reflection in the energy range from 6 keV to 25 keV. An evacuated flight tube reduces air absorption effects. The 2D imager is synchronized to the 10 Hz pulse train structure and integrates over the full bunch train. The 1D Gotthard detector operates in pulse resolved and is triggered with a maximum of 120 exposures at 556 kHz in synchronization with the X-ray bunch train and set to an exposure time of 220 ns, hence measuring every fourth pulse when operating at 2.25 MHz repetition rate.

**Figure 5:**
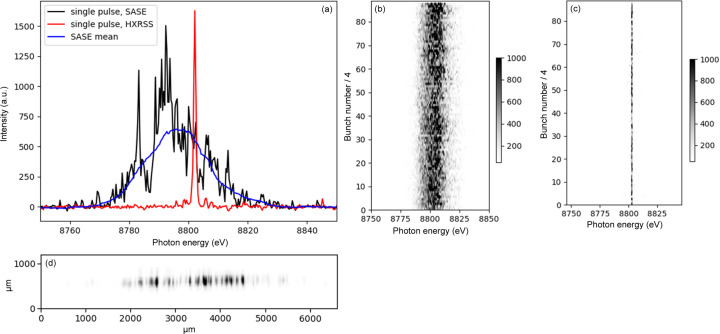
(*a*) Spectral distribution for a single pulse (black), SASE mean (blue) and single-pulse HXRSS measured with the Gotthard-I 1D detector. Spectral distribution plotted over the full pulse train of 350 bunches (2.25 MHz), displaying only every fourth pulse for (*b*) SASE and (*c*) HXRSS. (*d*) 2D measurement of a single pulse SASE spectrum using a YAG screen and a camera.

Fig. 5 ▸ shows an example of the spectral distribution of the EuXFEL X-ray beam measured using the C(220) reflection of a single-crystal diamond with a bending radius of approximately *R* = 90 mm. The incoming X-rays had a photon energy of 8.8 keV and a spot size of 0.5 mm × 0.5 mm. In Fig. 5 ▸(*a*), the spectral distribution of a single SASE pulse (black) and the mean SASE spectral distribution acquired over several minutes (blue) aquired with the Gotthard detector is shown. The red line shows a single pulse generated in the HXRSS mode. The spectral bandwidth of such a pulse is around 10^−4^, *i.e.* comparable to that of a monochromated beam using an Si(111) double-crystal monochromator (Liu *et al.*, 2023 ▸). In Fig. 5 ▸(*b*), the spectral distribution of the SASE beam over a single pulse train with 350 bunches is shown as a waterfall plot. Due to the limited repetition rate of the Gotthard-I detector, every fourth pulse is recorded. The same is shown in Fig. 5 ▸(*c*) for a pulse train in HXRSS mode. Fig. 5 ▸ (*d*) displays an example of the 2D distribution of a single SASE pulse acquired by the 2D YAG imager.

When the low-divergence (<2 µrad), polychromatic SASE beam impinges on the bent crystal the angle of incidence θ varies over the footprint of the beam. Therefore, the Bragg condition λ = 2*d*sinθ for reflecting lattice planes with spacing *d* is fulfilled for different wavelengths (λ) depending on the position on the crystal. Thus, the beam is dispersed in the scattering plane according to the photon energy and can be distinguished by position-sensitive photon detection. The geometric energy resolution per pixel δ*E*, here denoted pixel resolution, of the recorded spectrum depends on the pixel size Δ*p* of the detector and the crystal-to-detector distance *L*, and is given by 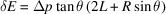 (Zhu *et al.*, 2013 ▸; Boesenberg *et al.*, 2017 ▸). For 9 keV, this leads to a theoretical value of δ*E* = 0.34 eV for the C(220) reflection with Δ*p* = 50 µm, *L* = 1 m and a bending radius *R* ≃ 90 mm. Experimentally, we have determined an energy resolution δ*E* = 0.35 eV for ∼9 keV incident photon energy, using the Cu *K* absorption edge as a reference. Depending on the photon energy and crystal reflection, the geometric energy resolution varies between 0.1 eV and 1.4 eV.

The experimental procedure to measure the spectrum is straightforward, but comprehensive understanding of the measured intensity distribution in the context of diffraction and wave properties requires theoretical efforts. The effect of the strong bending on the diffraction behavior and the consequent limitations of the achievable energy resolution determined by the setup have been investigated by theoretical modeling (Kaganer *et al.*, 2020 ▸, 2021 ▸; Samoylova *et al.*, 2019 ▸; Petrov, 2022 ▸). For the thin and strongly bent crystals used in the MID setup, dynamical diffraction effects were found to be negligible.

**Figure 6:**
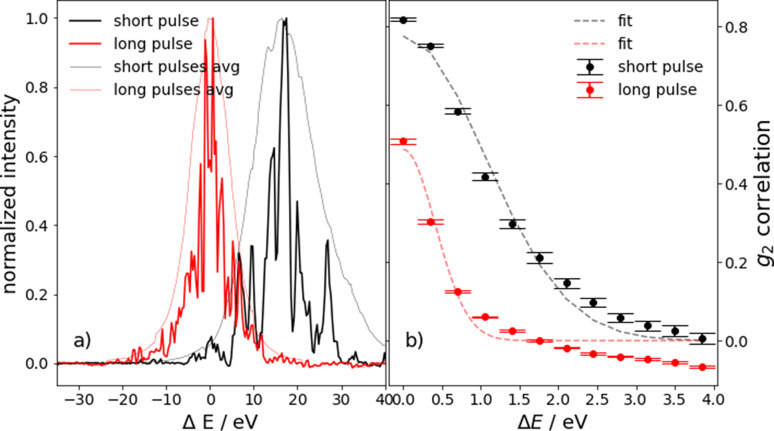
(*a*) Spectral distribution of a single-pulse SASE beam (9 keV) for different pulse durations achieved by a variation in the compression of the electron bunch. The black curves are slightly shifted from the central energy for better visibility. (*b*) Average of the *g*_2_ correlation function for the two different pulse characteristics shown in (*a*). A total of 17 360 spectra are used, and the fits are obtained by fitting a Gaussian peak function.

Due to the connection between temporal and spectral characteristics of electromagnetic waves, the pulse duration and energy bandwidth of the SASE spikes are inversely correlated. For a Gaussian-shaped X-ray pulse (SASE spike), the minimum duration is given by Δ*E*τ_0_ = 1.82 eV fs (Diels & Rudolph, 2006 ▸). Here, Δ*E* is the spike width in energy and τ_0_ is the pulse duration. Hence, for sufficiently well resolved SASE spectra, conclusions on the pulse duration may be drawn from the energy distribution recorded by the spectrometer (Inubushi *et al.*, 2012 ▸; Lutman *et al.*, 2012 ▸). The simplicity of the analysis methods in principle allows for almost real-time estimations of the pulse duration (Serkez *et al.*, 2019 ▸; Petrov, 2022 ▸). In Fig. 6 ▸(*a*), an example of spectra recorded for two different compression settings for the electron bunches in the accelerator leading to different pulse durations is shown.

Since the pulse duration is inversely proportional to the width of the SASE spikes, the shorter pulses have wider spectral features. By calculating the *g*_2_ correlation function (1), 

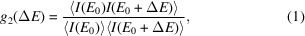

we can estimate the energy width of the peaks in the spectra (Petrov, 2022 ▸).

Here, Δ*E* is the photon energy difference from *E*_0_, where the brackets denote the averaging over *E*_0_ in a single spectrum. In order to consider inhomogeneous intensity distributions in the spectrum, the width of a region of interest is chosen to be 4 eV, where the average intensity varies by less than 2% for the *g*_2_ calculation. Fig. 6 ▸(*b*) shows the corresponding *g*_2_ correlation functions to the spectra in Fig. 6 ▸(*a*) and the fits used a Gaussian peak function. The fits for the full width at half-maximum (FWHM) of a Gaussian peak in Fig. 6 ▸(*b*) estimate values for the characteristic spike width to 0.98 eV and 2.48 eV for the long and short pulses, respectively. This indicates minimum pulse durations of 1.87 and 0.73 fs, respectively, according to the relation described above. These values are extremely small. For the longest pulses, the width of the SASE spikes (0.98) is comparable to the pixel resolution of the detector. For the shortest pulses, we found a spike width of 2.48 eV which is significantly larger than the pixel resolution. Still, the corresponding pulse duration of 0.73 fs is about a factor of eight smaller than that found in the experiment by Trost *et al.* (2023 ▸) using the same compression settings of the linac. We conclude that a significant ambiguity persists in how spectroscopic data can be used to determine the absolute pulse duration. A significant deviation of the real pulse duration from the estimations of the Fourier-transform-limited pulses has also been described by Makita *et al.* (2015 ▸). Since only the absolute intensity of the electromagnetic field is measured by the detector, as in any scattering experiment, the data lack information on the phase of the scattered wave. Hence, the correlation analysis assumes (1) a Gaussian distribution of energies over the SASE bandwidth and (2) no energy chirp in the electron beam. The second condition in particular is not necessarily fulfilled, as the variation in the pulse duration was achieved by variation of the electron bunch compression. In the presence of a pulse chirp, the bandwidth-pulse duration relation reformulates to Δ*E*τ_0_ = 1.82(1 + α)^1/2^ (eV fs) (Diels & Rudolph, 2006 ▸). Assuming a pulse duration of 6.2 fs (Trost *et al.*, 2023 ▸) for the short pulses, we obtain α = 70. Our estimated α value is significantly larger than those determined by Makita *et al.* (2015 ▸). For the standard long pulse duration, we do not have another independently obtained value for the pulse duration.

## 3. Discussion

The DES has proven extremely valuable for alignment and characterization of the X-ray beam at MID. The bent-crystal spectrometer provides valuable information for characterization and tuning of special modes such as HXRSS, two color mode or short-pulse operation.

The currently used line detector Gotthard-I installed in the spectrometer is incapable of measuring at the full repetition rate of EuXFEL (4.5 MHz). An upgraded version of Gotthard is under development at Paul Scherrer Institute (Villingen, Switzerland) (Zhang *et al.*, 2018 ▸) with implementation planned at MID in the near future to overcome the issue of missing bunches in the spectrometer data. The bent-diamond-crystal spectrometer showed no deterioration under the fierce beam conditions of up to 1000 bunches per train. In addition, for typical beam parameters such as 9 keV and the standard pulse duration, the spectrometer resolution is currently limited to the characterization of short pulses by the pixel size of the detector, rather than limited by the diffraction properties of the spectrometer crystal. Still, some uncertainty persists in how to relate the spectral fine structure to the pulse duration and we find a discrepancy between results obtained using different methods. As the absolute energy calibration of the spectrometer is a function of beam position, size and detector position, which typically vary from experiment to experiment, we currently mainly operate with a relative energy calibration. There are efforts ongoing to allow for an insertion of a thin diamond crystal upstream in the photon tunnel to generate a diffraction extinction in the spectrum (notch) to facilitate absolute energy calibration (Grech *et al.*, 2024 ▸).

As mentioned in the *Introduction* [Sec sec1], full characterization of the wavefront on single pulse level for different X-ray beam parameters would lead to significantly better understanding of the beam properties. For this, high-resolution megahertz imaging capabilities would be required at EuXFEL (Weitkamp *et al.*, 2005 ▸). For imaging of the divergent beam in the far field and for high-resolution SAXS experiments, we foresee a new multiple detector stage setup at MID, different from the DES.
